# A Monitoring Framework with Integrated Sensing Technologies for Enhanced Food Safety and Traceability

**DOI:** 10.3390/s22176509

**Published:** 2022-08-29

**Authors:** Antonio Vincenzo Radogna, Maria Elena Latino, Marta Menegoli, Carmela Tania Prontera, Gabriele Morgante, Diamantea Mongelli, Lucia Giampetruzzi, Angelo Corallo, Andrea Bondavalli, Luca Francioso

**Affiliations:** 1 Institute for Microelectronics and Microsystems, National Research Council of Italy (CNR-IMM), Campus Ecotekne, Via per Monteroni s.n., 73100 Lecce, Italy; 2 Department of Innovation Engineering, University of Salento, Campus Ecotekne, Via per Monteroni s.n., 73100 Lecce, Italy; 3 Resiltech s.r.l., Piazza N. Iotti, 25, 56025 Pontedera, Italy

**Keywords:** Industry 4.0, Agriculture 4.0, food traceability, ICT for sensor networks, Internet of Things, model based system engineering, glyphosate low-cost detection

## Abstract

A novel and low-cost framework for food traceability, composed by commercial and proprietary sensing devices, for the remote monitoring of air, water, soil parameters and herbicide contamination during the farming process, has been developed and verified in real crop environments. It offers an integrated approach to food traceability with embedded systems supervision, approaching the problem to testify the quality of the food product. Moreover, it fills the gap of missing low-cost systems for monitoring cropping environments and pesticides contamination, satisfying the wide interest of regulatory agencies and final customers for a sustainable farming. The novelty of the proposed monitoring framework lies in the realization and the adoption of a fully automated prototype for in situ glyphosate detection. This device consists of a custom-made and automated fluidic system which, leveraging on the Molecularly Imprinted Polymer (MIP) sensing technology, permits to detect unwanted glyphosate contamination. The custom electronic mainboard, called ElectroSense, exhibits both the potentiostatic read-out of the sensor and the fluidic control to accomplish continuous unattended measurements. The complementary monitored parameters from commercial sensing devices are: temperature, relative humidity, atmospheric pressure, volumetric water content, electrical conductivity of the soil, pH of the irrigation water, total Volatile Organic Compounds (VOCs) and equivalent CO${}_{2}$. The framework has been validated during the olive farming activity in an Italian company, proving its efficacy for food traceability. Finally, the system has been adopted in a different crop field where pesticides treatments are practiced. This has been done in order to prove its capability to perform first level detection of pesticide treatments. Good correlation results between chemical sensors signals and pesticides treatments are highlighted.

## 1. Introduction

The agri-food industry plays a crucial role in people’s safety. Food quality directly impacts on the health of the individual, as widely proved by each significant food crisis. Moreover, food production generates environmental repercussions that indirectly affects the quality of citizens’ life. Suffice it to say that food production and processing accounts for about one quarter of world carbon emissions [1]. A key role in the production chain is represented by the environmental factors surrounding the farming process: pollution in the air, soil and water affects the goodness and, in general, the quality of the cultivation. One of the more discussed sources of pollution in the modern agricultural practice, is represented by the improper use of pesticides. The absorption of high quantities of chemicals in agricultural products has been proved to be a risk for human health [2]. The interest of the scientific community and governments, regarding the adoption of substances like glyphosate or chlorpyrifos, is growing over time. This leaves space for controversies, among the regulatory agencies, that persist nowadays. For example, there are 72 pesticides admitted for open-air agriculture in the USA, that are banned or in the process of complete phase out in the EU [3]. In particular, glyphosate is listed by the International Agency for Research on Cancer (IARC) as a carcinogenic hazard to humans. It is also known to form highly stable complexes with transition metals. For this reason it is a high-risk element for different matrices, such as environmental (soil and water) and food [4]. According this regulation framework, food companies undertakes paths of transparency and sustainability in food production [5], leveraging on technology-based traceability systems. Today traceability, is considered as a new quality index in the food industry [6], resulting as a tool able to increase the performance of the entire food supply chain. Traceability systems make possible to monitor the environmental impact of lifecycle processes by linking the relevant environmental information to the traced products [7]. According to [8], a generic traceability system is composed of three main components: “Identification, database and information flow”. Among the data acquisition solution (identification component), tagging technologies were the widespread [9,10], such as optical codes or RFID [11,12]. If the tagging technologies are able to follow the food product along the supply chain, other sensing technologies are more suitable in monitoring the quality of the environment. Ref. [13] suggest several smart farming technologies for environment monitoring (e.g., RGB cameras, LIDAR sensors, ToF cameras, thermal cameras, NDVI sensors, soil moisture sensors) that are capable of collecting information about the food quality. The adoption of sensor-based monitoring systems can limit the manual data entry by supply chain operators, decreasing efforts and increasing the efficiency of forward and backward traceability. In this context various networks and devices were connected forming a global cyberspace which is connected, in turns, to the “natural space via various cyber physical interfaces like sensors to form a global cyber physical system” [14]. Ref. [15] propose a blockchain-based solution for agri-food traceability in order to assure the conformity with HACCP principles and requirements during production, logistics and stockage of a product. Ref. [16] propose a monitoring system, based on IoT, RFID, sensing, and cloud technologies, capable of ensuring food quality and safety also in the presence of HACCP specifications. Ref. [17] describe a traceability system based on the integration of RFID, wireless sensor networks, and data mining, that aims to identify an e-pedigree for kimchi product. Ref. [18] discuss a traceability system based on blockchain and NFC technology, capable of providing transparency and security in the food supply chain. A blockchain-based wine traceability system was proposed also by [19]. To the best of our knowledge, there is a lack of research studies that investigate the possibility to adopt environmental sensing technologies to collect data about the quality and healthiness of the cropping environment and use it for traceability purposes. Specifically, information related to the contamination of cropping environment with herbicides or substances harmful to humans, is of great importance and generates a wide interest for regulatory agencies and final customers. To this aim, the glyphosate [N-(phosphonomethyl) glycine] contamination monitoring represents a good candidate for a research activity, leading to a new generation of low-cost and selective sensors. Therefore, the current gold standard detection methods of glyphosate in water and soil are expensive techniques that require specialized personnel for tests, as, for example the liquid chromatography (LC) [20], the gas chromatography/mass spectrometry [21] or other methods. In order to overcome the above identified gap, the present study proposes a monitoring framework for food traceability that, leveraging on sensing technologies, is capable of monitoring the cropping environment, giving insight about the genuineness of food. Through the measurement of environmental and chemical parameters for air, water and soil, the proposed framework enrich the traceability data with information about the quality and healthiness of the cropping environment. In general, Internet-of-Things-based frameworks are nowadays a common practice for precision agriculture both in research projects and commercial solutions. What differentiates our work in respect to the state of the art is the adoption of a proprietary and automated system for glyphosate detection. It is useful for a first level monitoring of farming areas towards off-target contamination during herbicides application. Indeed, a critical problem for this kind of crops is the chemicals drift from neighboring properties due to wind and other atmospheric conditions. This aspect can be of particular interest when conventional farming techniques are applied in the vicinity of organic farms with possibility of crops contamination. Since this represents both an environmental damage and a serious economic loss [22], the introduction of a fast, fully automated, and in situ screening device, for the estimation of the concentration of glyphosate in air, can be a valuable tool for the quality monitoring of agriculture productions.

## 2. Proposed Monitoring Framework for Food Traceability

The primary case study is focused on a company involved in the olive farming activity located in Italy, Apulia. The extra virgin olive oil (EVOO) is a well-known gastronomy element considered as one of the basis of the Mediterranean diet. The high levels of antioxidants and monosaturated fats support the dietary control of the LDL cholesterol, increasing the HDL cholesterol. The monitoring parameters for the selected crop are glyphosate droplets sampled from air, atmospheric pressure and air temperature, soil electrical conductivity, volumetric water content, irrigation water pH, air equivalent CO${}_{2}$ concentration, and total volatile organic compound. They have been proposed for analysis by the research team of the *GoodForYou!* public funded project. Several scientific evidences report the correlation between the chosen monitoring parameters and organoleptic properties of produced oil and vegetables. Regarding the soil contamination by heavy metals, lead, mercury, cadmium and arsenic are toxic to humans and cause more serious problems when soil is contaminated; soil contamination can be detected after a long exposure time and early detection of contamination is very important in the recovery treatment of soil. Ref. [23] demonstrates that the electrical resistivity of contaminated soil specimens is lower than those of non-contaminated specimens. In contaminated soils, electrical resistivity and permittivity could be employed as alternative methods for determining harmful elements. The impact of temperature and air relative humidity (RH) on olive trees growth is also important and affects the olive plant growth. Ref. [24] reported that a high air temperature (37 ${}^{{}^{\circ}}C$) reduces plant dry matter accumulation only when the temperature of the root medium was also high (37 ${}^{{}^{\circ}}C$). When the temperature of the root medium was (25 ${}^{{}^{\circ}}C$), the inhibitory effect of high air temperature on plant growth was not observed. These results suggest also that high temperatures in the root of the olive tree inhibits the potassium transport. Among the selected parameters, the monitoring of CO${}_{2}$ related to respiration in olive trees is considered of great importance and implemented with a metal oxide-based gas sensor (Sensirion SGP30), that supply information on equivalent CO${}_{2}$ concentration and total volatile organic compounds (TVOC), useful for identification of environmental contamination from humans activities and treatments. About the respiration during the fruit setting periods, according to [25], approximately the 30% fraction is related to plant growth and 70% to maintenance. The authors provide an independent assessment of the plant organ composition effects on the total tree respiration and may be in our case integrated in a general maintenance process of the trees.

**Figure 1:**
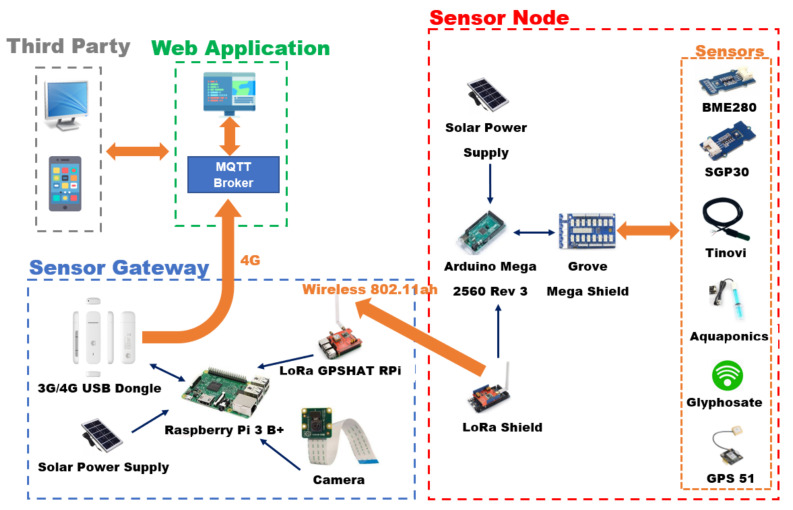
Simplified architecture diagram of the proposed device.

**Figure 2:**
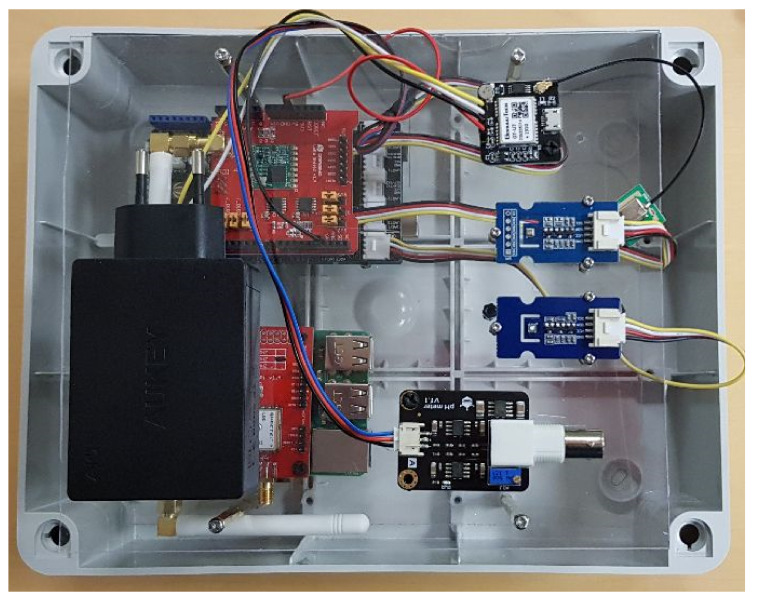
Photo of the commercial devices during assembly. The depicted devices are housed in a water tight enclosure along with the glyphosate detection system.

Regarding the implementation, the framework consists of the sensor nodes (SN), the gateway (GW) and the web server (WS). Hardware and software components of the entire architecture have been selected, guaranteeing a good trade-off between costs and performances. The presented prototype also integrates a custom-made and automated fluidic system, for a first level monitoring of farming areas towards off-target contamination during glyphosate application. In each farmer’s field were placed one SN and one GW. Moreover, several sensors able to monitor the parameters derived from the requirements analysis were chosen according the following requirements: operating temperature (from −40 ${}^{{}^{\circ}}C$ to 60 ${}^{{}^{\circ}}C$), small size and low weight, portability, ease of maintenance and replacement, self-sufficiency in energy, low cost, low detection times, standard communication interfaces and protocols. Figure 1 and Figure 2 show a simplified diagram of the hardware architecture and a photo of the realized prototype during assembly, respectively. The adopted solution is capable of monitoring the cropping environments, even in unfavorable conditions (rain, humidity, etc.), avoiding tampering due to natural or malicious causes.

The LoRa wireless protocol has been used as communication link between SN and GW. This protocol implements a single-hop link, ensuring greater control over latency: the SN devices, as the environmental sensors, are arranged according to a topology that is typically defined as “star-of-stars” with the GW in transparent bridge mode, transmitting messages from the end nodes to the web application.

The SN transmits, through LoRa connection, a payload in JSON format representing values and unit of measures of parameters detected by the sensors. The data package structure is an array containing the unique ID generated with 128-bit UUID algorithm, the measured parameter, the unit of measure name and finally the parameter value (valuer). Table 1 summarizes the hardware components of the SN. The GW device is composed by two hardware components:

- LoRa-GPS HAT for Raspberry Pi (Dragino Technology Co., LTD.): expansion board for Raspberry equipped with the long-range LoRa modem;
- Raspberry Pi 3 Model B+ (Raspberry Pi Foundation) embedded computer.

The data transmission from the Raspberry Pi 3 to the broker is performed by a 3G/4G USB dongle router (Huawei E3372 4G). It is able to supply a 4G LTE internet connection.

**Table 1:** Hardware components of the SN.

Component	Description	Parameter	Output
Dragino LoRa	LoRa shield for Raspberry Pi as communication link between the SN and the GW	-	-
Arduino Mega2560	Microcontroller board based on the ATmega2560. In the SN it was powered by a solar panel and was programmed using Arduino Software (IDE).	-	-
Grove—Mega Shield	Extension board for Arduino Mega 2560	-	-
BarometerQualitySensor_Grove BME280	Precise and fast sensor able to detect humidity, atmospheric pressure and temperature.	Humidity, Atmospheric Pressure, Temperature, Altitude	Digital
GasQualitySensor_Grove SGP30	Gas and humidity sensor.	eCO${}_{2}$ (equivalent calculated carbon-dioxide), TVOC (Total Volatile Organic Compound)	Digital
Glyphosate proprietary detector	The device is able to estimate the glyphosate concentration in the air.	Glyphosate presence	Digital
SoilMoistureSensor_Tinovi PM-WCS-3-I2C	Sensor able to detect dielectric permittivity, soil temperature, degree of water saturation in the soil and electrical conductivity	Dielectric permittivity, Temperature, Degree of water saturation, Electrical conductivity	Digital
WaterQualitySensor_D&F Aquaponics DF/1100711	Sensor able to detect the Phof the water.	pH	Analog
GPS Sensor	Sensor able to detect the geographical coordinates	Coordinates	Digital

As already mentioned, an innovative feature, in respect to the state of the art, resides in the custom-made and automated fluidic system for glyphosate detection. The sensing device is an electrochemical sensor based on MIP technology and it allows to detect and estimate the concentration of glyphosate in the air. MIP materials expose to liquid sample specific imprinting sites which can match with the target molecule in shape, size and functional groups [26]. This property allows MIPs to specifically bind to target molecules, with a good selectivity compared to other chemical compounds. From a holistic point of view, the sensors selection has been performed with a special attention to low cost, easy interconnection and electronics front-end requirements. In particular, this device could be very beneficial to the vulnerable production of olives in the south of Italy, threatened by the spreading of *Xylella fastidiosa* disease occurred in the last years. The adoption of the electrochemical MIP sensing technology, which is known to be suited for liquid matrices, lead to a precise sequence of tasks in order to properly carry out the detection of glyphosate in environmental air. As detailed in this section, the injection and exhaustion of various solutions with accurate control of quantity and the pumping of external air in the electrochemical cell must be ensured in order to perform a proper unsupervised measurement with this sensor. In particular, the following challenges have been addressed:

1. The complete automation of injection (and exhaustion) of solutions and pumping of external air in the electrochemical cell;
2. The adoption of a small, low-power and low-cost potentiostat for the cyclic voltammetry measurements instead of big, power hungry and expensive potentiostats normally used for laboratory practice.

**Figure 3:**
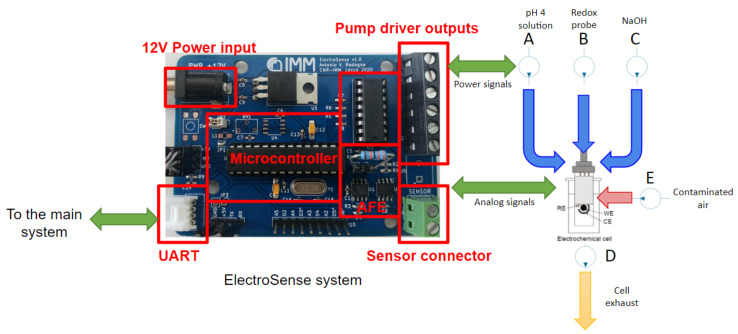
Photo of the ElectroSense mainboard (**left**) and picture of the fluidic circuit (**right**).

Thus, a single electronic device has been properly designed in order to implement all the mentioned tasks for unsupervised on-field measurements. The architecture of the glyphosate detector interface and the fabricated silicon sensor are depicted in Figure 3.

The system includes 3 main blocks:

- **The electrochemical cell:** it consists of an electrochemical sensor fabricated on alumina substrate with thin film microfabricated electrodes located in a glass cell for automatic measurements cycles. The molecularly imprinted polymers (MIP) technology has been chosen thanks to the high selectivity, sensitivity, and ruggedness performances of the sensing materials [27]. The measurement is performed in phosfate buffer with the addition of a mixture of 5 mM potassium ferro/ferricyanide as redox probe, after an incubation period of 10 min in the analytical solution. As the glyphosate is present in the analytical solution, its molecules bind to the cavity of the MIP electrode, thus inducing a reduction of the electrochemical active area of the working electrode. The subsequent measurement with the addition of a redox probe allows to evaluate the active area of the working electrode and, thus, the concentration of glyphosate is determined through indirect measurement. A TC6 electrochemical cell (BVT Technologies, a.s.) has been used for the automated system;
- **The ElectroSense mainboard:** the custom-made electronic potentiostat which includes the analog front-end (AFE) for the potentiostatic read-out of the sensor, the digital control unit, i.e., the microcontroller, and the drivers for the diaphragm pumps;
- **The fluidic circuit:** it implements, through the pumps A,B,C,D and E depicted in Figure 3, the solutions handling for the proper analysis of sampled air in the sensor cell. The fluidic circuit comprises small tanks for the proper operation of the sensor. The adopted solutions are: (1) a pH 4 solution for glyphosate rebinding (analytical solution), (2) phosphate buffer saline (PBS) with the addition of the redox probe, and (3) NaOH solution necessary for the regeneration of the sensor.

The detection system is operated 2 times a day, with a 12 h interval: the morning at 6:00 and the evening at the 18:00. The adopted electrochemical technique for the sensor read-out measurement is the cyclic voltammetry. This technique consists of both stimulating the sensor with an input voltage sweep, acquiring the output current from the sensor. A complete read-out cycle, previously optimized to enhance the sensitivity of the sensor, consists of:

1. 10 voltammetry cycles performed with PBS and redox probe;
2. Incubation of the sensor (10 min) in the external sampling solution (pH4 buffer solution);
3. 10 voltammetry cycles in PBS + redox probe.

The air sampling pump continuously works for 20 min, allowing the bubbling of about 10 L of environmental air in the pH4 sampling solution. The measurements performed in (1) and (3) steps are compared in post-processing stage in order to obtain the estimation of glyphosate concentration in the surrounding environment. In particular, a reduction of the oxidation and reduction peaks of the redox probe is visible in presence of glyphosate. The entity of the reduction depends on the glyphosate concentration. After the completion of the measure, the detector’s operation is concluded by refilling the cell with NaOH, performing 10 voltammetry cycles in order to detach any glyphosate bound to the electrode and regenerate the electrode for the subsequent measurement. All of the aforementioned operations are executed without human supervision, thanks to the ElectroSense embedded control unit. Both the sensor and the ElectroSense board have been properly characterized in advance and validated with laboratory equipment.

**Figure 4:**
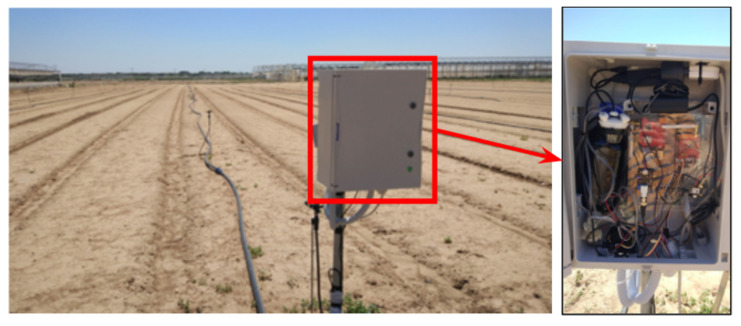
Photo of the monitoring prototype installed in a test field.

The described system, depicted in Figure 4, has been assembled in a water tight enclosure. The assembled system of Figure 4 has been validated in a real crop field. Regarding the cost of the ElectroSense device, a comparison can be done with commercial potentiostats. These can be found on the market in a very broad range of prices, typically from several hundreds of EUR to several thousands of EUR. This cost is justified since, being general purpose equipment for laboratories, they can run a multitude of electrical measurements with additional data processing and filtering through sophisticate software. On the contrary, our device is specifically optimized for a single application, i.e, cyclic voltammetry and fluidic automation. The cost of the printed circuit board (PCB), plus the integrated circuits (the microcontroller, general purpose operational amplifiers and pump drivers) plus the on-board connectors is estimated to be about 15 EUR.

## 3. Experimental Results

The MIP sensor has been realized onto 500 μm thick alumina substrate with a circular chromium/gold (10/200 nm film thickness) working electrode (2 mm diameter) and a chromium/platinum (10/200 nm film thickness) pseudo-reference electrode [28].

**Figure 5:**
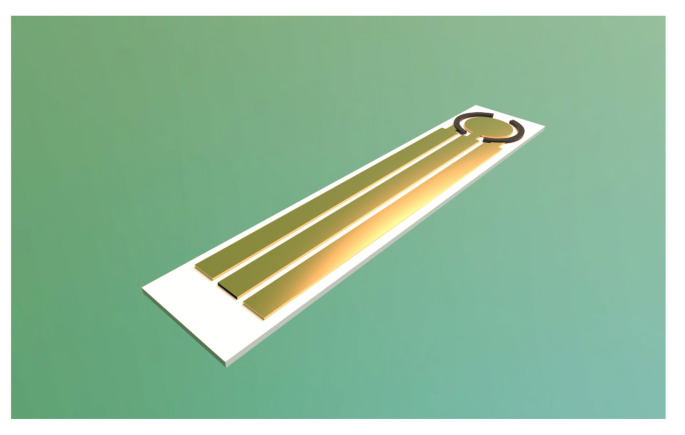
Rendered 3D model of the fabricated MIP sensor.

**Figure 6:**
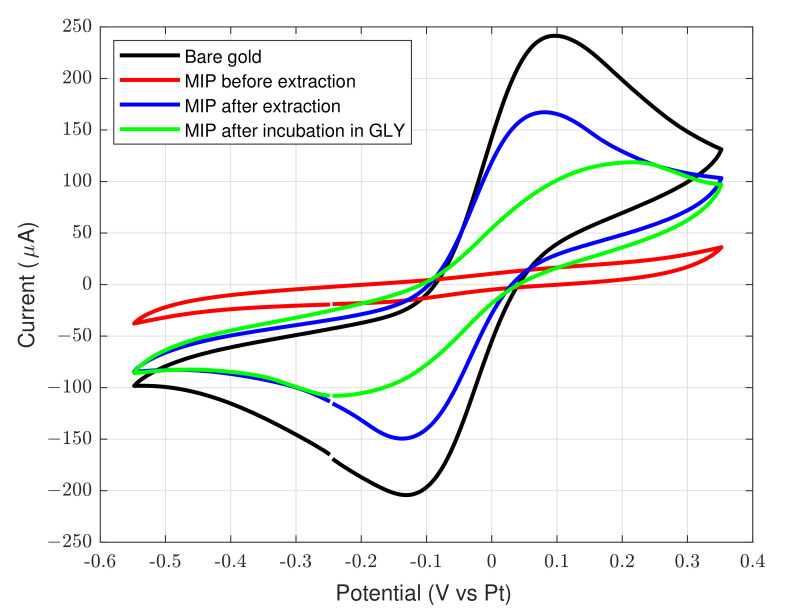
Cyclic voltammograms of differently modified electrodes in PBS + redox probe. (black) the bare gold electrode, (red) MIP modified gold electrode after elution, (blue) MIP modified gold electrode after elution, (green) MIP modified gold electrode with bound glyphosate. In the latter, the used concentration is 500 ng mL^−1^.

**Figure 7:**
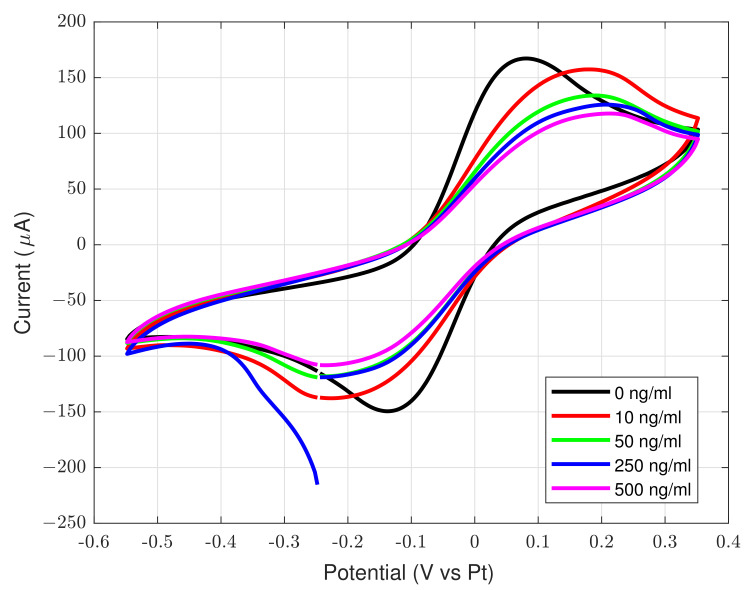
Cyclic voltammograms of MIP modified gold electrode in PBS + redox probe after incubation in analytical solution at different glyphosate concentrations.

A rendered 3D model of the realized device is depicted in Figure 5. The cyclic voltammetry curves of the different fabrication steps are reported in Figure 6. The current response of redox probe at bare gold electrode is the largest and peaks at −0.1 V (red peak) and 0.1 V (ox peak) are visible (referred to the integrated platinum electrode). After the pyrrole/glyphosate deposition the current is strongly reduced due to the formation of a non conductive polymeric thin film. The glyphosate extraction induces an increase of the peaks current as consequence of cavity formation. Finally, when the MIP is incubated with glyphosate, the cavities of the polymer layer are filled and the peak current decreases. A representative curve of the sensor, after incubation in a 500 ng L^−1^ solution, is reported in Figure 6 (green curve). The glyphosate detector has been preliminarly characterized in a controlled environment with different target concentrations from 10 ng mL^−1^ to 500 ng mL^−1^ considering the estimated spray concentration of the target molecule [29] and the air volume sampled from the pump (about 50 L) and bubbled in the rebinding solution with submerged sensor. The different voltammograms are reported in Figure 7. As expected, a reduction of the peak current is visible in the figure with the increase of glyphosate concentration. The peak current values, at different glyphosate concentrations, are normalized in respect to the peak current at 0 ng mL^−1^. In this way, a normalized sensor response is obtained and it assumes 100% for 0 ng mL^−1^, 94% for 10 ng mL^−1^, 80% for 50 ng mL^−1^, 75% for 250 ng mL^−1^, and 70% for 500 ng mL^−1^. This behaviour is expected due to reduced surface of the working electrode exposed to redox probe after the glyphosate binding. The voltammograms reported in Figure 6 and Figure 7 have been performed by using a commercial potentiostat/galvanostat (Ivium Vertex One, Ivium Technologies B.V., Netherlands) in a two-electrode configuration with a platinum reference/counter electrode.

**Figure 8:**
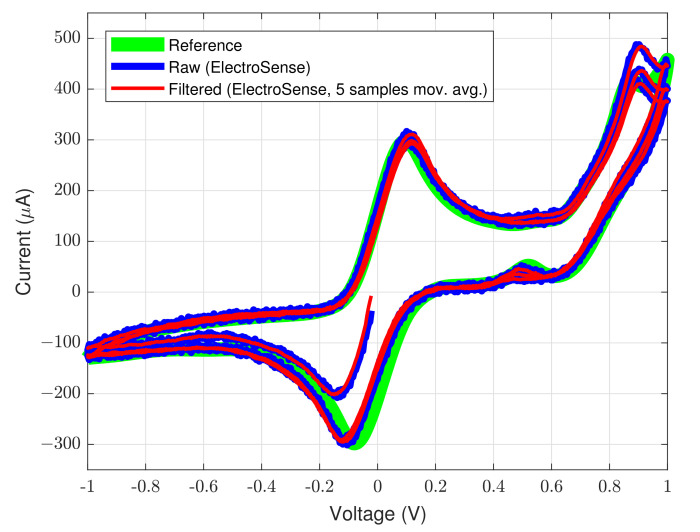
Reference voltammogram obtained from commercial instrument (green), raw voltammogram obtained from ElectroSense (blue), and filtered voltammogram from ElectroSense (red) obtained through a moving average filtering of the raw curve with a window of 5 samples.

In order to test the potentiostatic read-out functionality of the ElectroSense mainboard, 3 voltammetry cycles of a gold working electrode with a redox probe solution, have been carried out. The obtained curves are depicted in Figure 8. The figure shows the raw voltammetry (in blue color), obtained from the circuit and its filtered version (in red color). The applied voltage range goes from −1 V to +1 V and a scan rate of 100 mV s^−1^ has been used. In order to smooth the voltammograms obtained from the ElectroSense device, the raw measurements have been filtered through a basic moving average filter. The filtering has been performed in MATLAB environment by using a window of 5 samples. Moving average filters act as low-pass filters and they represent an easy-to-implement way to smooth the raw measurements from sensors since, the latters, are notoriously affected by disturbances such as electronic noise, environmental interferences, technological non-idealities, etc. The curves are compared with the ones from the commercial instrument and, as it can be noted by comparing the green curve (reference voltammogram from commercial instrument) and the red curve (filtered voltammogram from the ElectroSense mainboard) in Figure 8, there is a good matching between the obtained measurements. The automated system has been installed in 2 test fields for the parameter monitoring and experimental validation.

**Figure 9:**
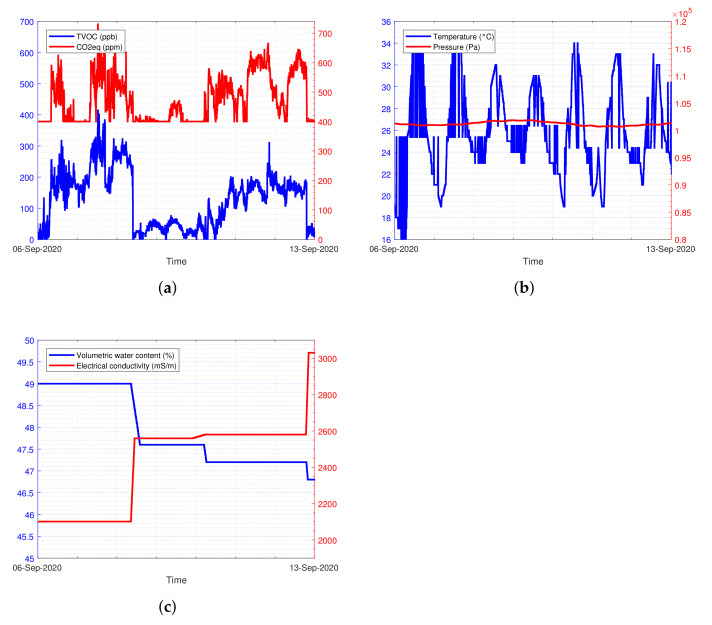
Experimental data waveforms collected from the test field, during the olive farming activity, in a time interval of 1 week: (**a**) TVOC and eCO${}_{2}$ measured in ppb and ppm, respectively; (**b**) temperature and pressure measured in ${}^{{}^{\circ}}C$ and Pa, respectively; (**c**) volumetric water content (VWC) and electrical conductivity measured in % and mS/m, respectively.

The assembled system of Figure 4 has been validated in a real crops field. Figure 9 depicts the experimental data waveforms collected from a field in a time interval of 1 week.

## 4. Discussion

Figure 9 reports the experimental data waveforms collected from an Apulian farmer field. The data shows valuable insights about olive plant growth and development. Depending on the benefits resulting from their knowledge, the monitored parameters can be categorized in 2 groups:

- **A group (consumer-oriented):** environmental temperature, TVOC, CO2eq, glyphosate;
- **B group (producer-oriented):** volumetric water content (VWC), electrical conductivity (EC), pressure, pH.

Parameters in A group could enhance the product’s perception, from the customers’ point of view, about the quality of the product. Therefore them are useful to increase the awareness about the genuineness and the health-related aspects about crop methods. Parameters in B groups, like the environmental temperature, could give predictions about flowering, vegetative growth, and fruit growth in olive trees [30]. In particular, awareness about the environmental temperature allows the farmer to optimize the crop management strategies, in order to maximize production during the ongoing phenomenon of climate change and to increase cropping sustainability [31]. Since the effects of different temperatures and, in general, weather conditions, directly impact the aromatic compounds, pigments and phenolic compounds, the monitoring of environmental temperature gives useful insights about the quality of olive oil [32].

Regarding the environmental air quality measurements, whose data are plotted in Figure 9a, it is known that agricultural crops are injured when exposed to high concentrations of various air pollutants and toxic substances [33]. Injuries comprise visible markings on the foliage, reduced growth and premature death of the plant. The entity of the injury depends on many factors, e.g., the concentration of the pollutant, the length of exposure, the plant species and its stage of the plant development and other environmental factors [34]. In order to evaluate the environmental air quality of the investigated crops, the TVOC (total volatile organic compounds) is measured through the realized prototype: this parameter has been introduced to give a practical and cost-effective indicator for environmental measurements of volatile organic compounds (VOCs). It takes into account the sum of all the contributions of the measured concentrations of VOCs [35]. It could be considered as an indicator of the potency of a pollution to cause health effects. Unfortunately, the use of the TVOC in literature has not yet been standardized and various authors use different definitions [36]. In this study, the definition and the ranges given in [37] have been considered. It is important to point out that the analysis of the environmental air quality is aimed to make a simple and effective first level (yes/no) detection of treatments, intended as an additional experimental chemical sensing that can support a multivariate data analysis or a decision support system based on machine learning or deep learning techniques [38]. This justifies the adoption of a low-cost air quality sensor, i.e., the SGP30. The exact identification of the substances in the treatment is out of scope for the present study and it would require a more complex, large and expensive instrumentation for the analysis. An important phenomenon that can be analyzed, mainly through the adoption of an air quality sensor for TVOC detection, is the spray drift: during and after pesticides treatment, a substantial fraction of substances, existing as small droplets (aerosols), are suspended in the surrounding environment. It can be transported over short and long distances, causing environmental air pollution and this effect can be detected by our system, as reported below. The impact led by the volatilization process of such substances, can be influenced by temperature, relative humidity, air temperature, atmospheric pressure, wind velocity and irradiation [39]. Pesticides treatments also lead to emission from soil. This process involves the from soil surface and wind erosion of soil particles containing sorbed pesticides. The phenomenon is contributed by many factors, like chemical properties of the compounds, soil properties, weather conditions and agricultural practices. The realized prototype of the glyphosate detector has been adopted in real field experimentation notable for avoiding the glyphosate usage as standard procedure. All the obtained voltammetry curves are similar to the black curve (0 ng mL^−1^) of Figure 7, exhibiting a normalized response of about 100% at the 0.1 V peak of the redox probe. This indicates the absence of glyphosate contamination either from direct pesticide treatment or from spray drift. This information, particularly relevant for organic crops, could enrich the perceived quality of the product from the consumer’s point of view. In order to obtain a further step of validation, the realized framework has been used in a different crop field (sugarloaf lettuce), where pesticides treatments are adopted. The increase in the mean TVOC can be observed for a time window of about 3 days. During these time intervals, no other actions on the field, such as irrigations or fertilization, have been performed. These results prove that the proposed low-cost device can successfully identify the presence of spray treatments in the environmental air around around the monitored field and, if the glyphosate molecule is present, the combined analysis of the both sensor signal can strengthen the chemical classification and increase the accuracy of the method. This information could be used to increase the consumer’s awareness about the exposure to pesticides, the quality of the product and the overall sustainability of the monitored cropping.

**Figure 10:**
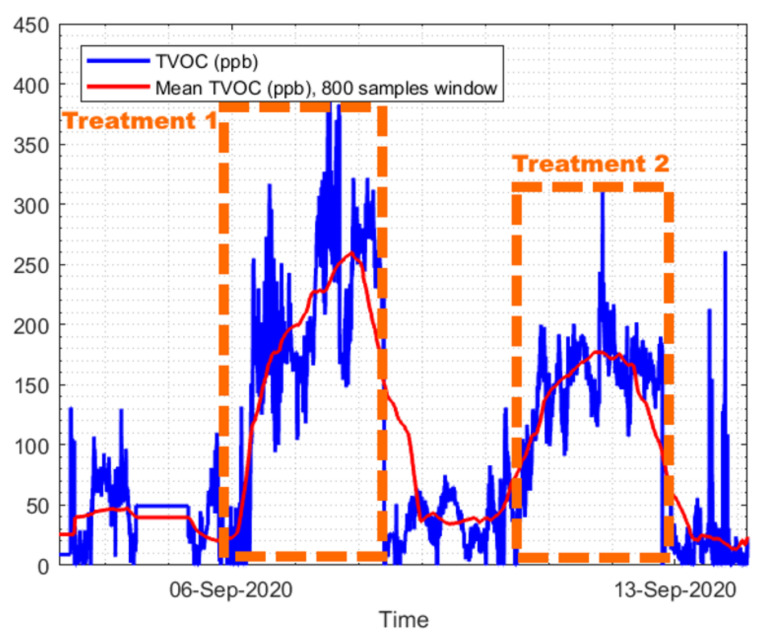
Collected TVOC samples (blue) and mean values (red), from the sugarloaf lettuce field, with identification of pesticides treatment.

In particular, the aim was the assessment of the possibility to detect the impact of pesticide treatment on the final product. Figure 10 shows the collected TVOC samples from this experimentation field. More specifically, the blue curve in the figure represents the raw TVOC measurements from the sensor, whereas the red curve is its smoothed version, obtained by filtering the raw data through a basic moving average filter with a window of 800 samples. The processing has been performed in MATLAB environment. The plot shows an increase of the mean TVOC value (red curve) in correspondence of the pesticides treatments reported in Table 2.

## 5. Conclusions

In this study, a novel and low-cost technological framework for food traceability, composed by commercial and proprietary sensors, has been proposed. The framework aims to the remote monitoring of air, water and soil parameters during the farming process. Specifically, it is capable of monitoring environmental temperature and relative humidity, pressure, volumetric water content and electrical conductivity of the soil, pH of the irrigation water, total VOC and equivalent CO${}_{2}$ in the environment. In addition, a proprietary device, for the first level detection of glyphosate contamination, has been realized. This device consists of an MIP-based electrochemical sensor, an analog front-end for control and signal read-out, and an automatic fluidic system. The framework has been applied and validated in a case study of olive producer company, proving its usefulness in traceability systems for agri-food productions. An additional experimentation has been made in a sugarloaf lettuce field in order to prove the system’s capability to first level detection of pesticides treatments. In this context, good correlation results, between chemical sensors signals (selective and non-selective types) and pesticides treatments, have been highlighted. Moreover, the correlation analysis has also been exploited in order to avoid unwanted contamination detection. The realized framework fills the gap of low-cost devices for monitoring cropping environments and pesticides contamination, offering a commercial opportunity for farmer and food companies involved in food quality path. The experimental activities of this framework proved its efficacy in food traceability. Thanks to its adoption, food companies, especially the ones involved in organic fruits and vegetables, could become capable of: (1) collecting data about the healthiness of the cropping environment and, (2) proposing, a traceability service through which intercepting the increasing target of conscious consumers, which aim to preserve environment and healthy during the food choice. The outcome of this benefit is an increase in competitiveness. The benefits can be enlarged to the supply chain operators, offering the possibility to increase efficiency in risk and responsibility management. Moreover, data represent a benefit for the final consumer allowing a conscious food choice.

## Figures and Tables

**Table 2:** Performed pesticides treatments, in the sugarloaf lettuce field, with mean TVOC detected peaks.

Treatment	Pesticides	Scheduled Date	Mean TVOC Detected Peak (ppb)
**1**	Boscalid 267,000 g/kg, Pyraclostrobin 67,000 g/kg, Indoxacarb 300,000 g/kg, Ethoxylated fatty alcohol 98,000 g/L	7 September 2020	250
**2**	B. Thur.Aizawai 500,000 g/kg, B. Thur.Kurstaki 500,000 g/kg	11 September 2020	180

## Data Availability

Not applicable.

## Abbreviations

The following abbreviations are used in this manuscript:

MIP	Molecularly Imprinted Polymer
VOC	Volatile Organic Compound
TVOC	Total Volatile Organic Compound
EVOO	Extra Virgin Olive Oil
SN	Sensor Node
GW	Gateway
WS	Web Server
AFE	Analog Front-End
VWC	Volumetric Water Content
